# Effects of uteroplacental insufficiency on growth-restricted rats with altered lung development: A metabolomic analysis

**DOI:** 10.3389/fped.2022.952313

**Published:** 2022-09-08

**Authors:** Merryl Esther Yuliana, Zheng-Hao Huang, Hsiu-Chu Chou, Chung-Ming Chen

**Affiliations:** ^1^International PhD Program in Medicine, College of Medicine, Taipei Medical University, Taipei, Taiwan; ^2^Faculty of Medicine, Christian University of Indonesia, Jakarta, Indonesia; ^3^Department of Pediatrics, School of Medicine, College of Medicine, Taipei Medical University, Taipei, Taiwan; ^4^Department of Anatomy and Cell Biology, School of Medicine, College of Medicine, Taipei Medical University, Taipei, Taiwan; ^5^Department of Pediatrics, Taipei Medical University Hospital, Taipei, Taiwan

**Keywords:** uteroplacental insufficiency, intrauterine growth restriction, lung development, metabolomics, radial alveolar count

## Abstract

**Background:**

Intrauterine growth restriction (IUGR) is among the most challenging problems in antenatal care. Several factors implicated in the pathophysiology of IUGR have been identified. We aimed to investigate the effect of UPI on lung development by identifying metabolic changes during the first seven days of postnatal life.

**Materials and methods:**

On gestation day 17, four time-dated pregnant Sprague Dawley rats were randomized to a IUGR group or a control group, which underwent an IUGR protocol comprising bilateral uterine vessel ligation and sham surgery, respectively. On gestation day 22, 39 control and 26 IUGR pups were naturally delivered. The rat pups were randomly selected from the control and IUGR group on postnatal day 7. The pups' lungs were excised for histological, Western blot, and metabolomic analyses. Liquid chromatography mass spectrometry was performed for metabolomic analyses.

**Results:**

UPI induced IUGR, as evidenced by the IUGR rat pups having a significantly lower average body weight than the control rat pups on postnatal day 7. The control rats exhibited healthy endothelial cell healthy and vascular development, and the IUGR rats had a significantly lower average radial alveolar count than the control rats. The mean birth weight of the 26 IUGR rats (5.89 ± 0.74 g) was significantly lower than that of the 39 control rats (6.36 ± 0.55 g; *p* < 0.01). UPI decreased the levels of platelet-derived growth factor-A (PDGF-A) and PDGF-B in the IUGR newborn rats. One-way analysis of variance revealed 345 features in the pathway, 14 of which were significant. Regarding major differential metabolites, 10 of the 65 metabolites examined differed significantly between the groups (*p* < 0.05). Metabolite pathway enrichment analysis revealed significant between-group differences in the metabolism of glutathione, arginine–proline, thiamine, taurine–hypotaurine, pantothenate, alanine–aspartate–glutamate, cysteine–methionine, glycine–serine–threonine, glycerophospholipid, and purine as well as in the biosynthesis of aminoacyl-tRNA, pantothenate, and CoA.

**Conclusions:**

UPI alters lung development and metabolomics in growth-restricted newborn rats. Our findings may elucidate new metabolic mechanisms underlying IUGR-induced altered lung development and serve as a reference for the development of prevention and treatment strategies for IUGR-induced altered lung development.

## Introduction

Intrauterine growth restriction (IUGR) is one of the most challenging issues in antenatal care. Occurring in 5–10% of pregnancies, IUGR is a major predictor of perinatal morbidity and mortality (1). IUGR refers to a fetus that does not develop to its full biological potential in intrauterine, confirmed by a documented low fetal growth rate. IUGR is a pathological condition in which the placenta fails to provide an adequate supply of oxygen and nutrients to the developing fetus, which is called placental insufficiency. As a result, fetal growth becomes stunted. Several factors implicated in the pathophysiological of IUGR have been identified. Heredity, maternal availability of nutrients, and environmental factors, combined with the capacity of the placenta to adequately transfer nutrients and oxygen to the fetus, and the endocrine modulation of these interactions, are basic determinants of IUGR (2). Nonetheless, impaired placentation, including uteroplacental insufficiency (UPI), remains the primary and most common etiology and factor involved in the pathophysiology of the IUGR (1, 3). When UPI occurs, the developing fetus is subject to hypoxia and malnutrition because of the compromised placental blood supply (4). During pregnancy, chronic nutritional or oxygen deprivation may cause fetal airway and lung anomalies. This may put the fetus at risk of respiratory distress and bronchopulmonary dysplasia (BPD) during the pregnancy. These structural abnormalities and lung function impairment may remain or even expand with age. IUGR can even cause lifelong lung symptoms and accelerate lung aging (5). Recent studies suggest that decreased vascular endothelial growth factor receptor (VEGF) expression contributes to the pathogenesis of BPD that pulmonary circulation growth and alveolarization are highly coordinated, as demonstrated by the finding that impaired angiogenesis impairs lung structure. Angiogenesis is essential for the development and regeneration of human tissues. VEGF, a key mediator of angiogenesis and vasculogenesis, is required for the normal development of many tissues, including lungs. VEGF, which is found on the basement membrane of epithelial cells in the fetal lung, is thought to play an important role in guiding the development of the newly formed capillary network within the lung. In the absence of VEGF, lung maturation, surfactant production, and blood vessel and alveolar hypoplasia occur. These tissue abnormalities and altered tissue VEGF levels have been observed in humans and animal models with pulmonary diseases of infancy, such as respiratory distress syndrome, BPD, and pulmonary hypoplasia. VEGF increase alveolar units. Treatment of neonatal rats with VEGF inhibitors and antiangiogenic agents decreased alveolarization, vascular growth and lung growth in infant rats, which was similar to the pulmonary histology of BPD. This suggests that impaired VEGF signaling may contribute to abnormal lung structure following neonatal lung injury (6, 7).

Metabolites are valuable biomarkers in cell physiology because they play key roles in biological pathways (8). Metabolomic analysis refers to the analysis of changes in low-molecular-weight metabolites produced during disease progression. Metabolomes respond rapidly to even the smallest stimuli, which make metabolomic analysis a powerful approach to assessing quantitative responses to stress and nutritional alterations (8). The analysis of IUGR rat lung tissues and the metabolic changes there in can aid in the identification of the underlying etiological mechanisms and potential biomarkers of IUGR as well as potential therapeutic targets for its treatment. These findings may lead us to speculate that UPI is associated with impaired lung development and will serve as a reference for human studies in future studies. We hypothesized that the pulmonary metabolomes of IUGR rats recorded during the first 7 days after their birth would reveal the differentiation of their lung patterns reflected in their histological outcomes. Therefore, we conducted this study aiming to investigate the effect of UPI on altered lung development by identifying metabolic changes during the first 7 days of postnatal life in the lungs of IUGR rats.

## Materials and methods

### Animal model and experimental groups

This study was performed in accordance with the guidelines provided by the Animal Care Use Committee of Taipei Medical University (Taipei, Taiwan). Time-dated pregnant Sprague Dawley rats (180–250 g, 6–8 weeks old, and at 14 days gestation) were purchased from BioLASCO (Taiwan). For 1week prior to the experiment, all the experimental rats were provided with food and water ad libitum and were housed in a room at a temperature of 20–25 °C and a relative humidity of 40–60%. A 12:12-h light–dark cycle was maintained. On gestation day 17, bilateral uterine vascular ligation and a sham surgical protocol were performed. Isoflurane is used to induce general anesthesia in animals. A midline laparotomy was performed to reveal the uterine horns and their blood vessels, and the fetus was counted before surgery. According to the literature, bilateral uterine vascular ligation sites were selected (9). The uterine blood vessels are tied in the middle of each uterine horn, allowing blood flow from both the iliac arteries and the ovaries to continue. This performance resulted in a higher fetal viability rate and a lower partial abortion rate in the test group. The animals in the control group were subjected to a sham procedure that did not involve ligation. Animals were given lidocane at the incision site after the uterus was reinserted into the abdominal cavity (9, 10). On gestation day 22, all the rat pups were naturally delivered. Within 12 h of birth, litters were within-group pooled and randomly redistributed to the newly delivered mothers to ensure comparability and to prevent differing litter sizes from affecting the study outcomes. After the pups were euthanized on postnatal days 0 and 7, the litters in both groups were culled to nine and four pups to ensure equal access to breast milk, respectively. On postnatal day 7, rat pups were randomly selected from each group, irrespective of sex, and their lung tissues were harvested for histological, Western blot, and metabolomic analyses.

### Lung histology

The lung sections were harvested from the right middle lobe to standardize the analyses. Each of the sections was fixed in 4% paraformaldehyde, dehydrated in alcohol, rinsed with xylene, embedded in paraffin, cut into 5-μm sections, and stained with hematoxylin and eosin. To assess the morphometry of the rats' lungs, pathological alterations in the lung tissues were examined using a light microscope. The radial alveolar count (RAC) in each tissue sample was measured to evaluate the structural development of the alveoli according to a method modified from Cooney et al. (11). In order to offset bias, in addition to adhering to the rules emphasized by them, we strictly select objects for the RAC only when the respiratory bronchioles are symmetrical and the number of alveoli is within 4–10 transected by the perpendicular line from the center of the respiratory bronchioles to the pleura. The mean septal thickness (MST) was measured as described by Gao et al. (12). Briefly, 5 portions of every section were randomly selected and captured at 200× magnification and then analyzed using a computerized image analysis system (Image-Pro Plus 5.1 for Windows; Media Cybernetics, Bethesda, MD, USA). The septal thickness was measured using lines (60 per field) drawn at 90 angles across the narrowest section of alveolar walls (to minimize the number of tangential sections). An average value and its standard deviation were calculated.

### Western blot analyses of PDGF-A and PDGF-B

The lung tissue samples (0.06 g) were homogenized in ice-cold buffer. Furthermore, it was trypsinized and rinsed with phosphate buffered saline (PBS) before resuspension (1,500 rpm, 7 min). After the PBS was aspirated, 100 μl lysis buffer (1% Nonidet P-40 (NP-40), 0.01 M deoxycnolic acid (Sigma-Aldrich, St. Louis, MO, USA), 0.1% sodium dodecyl sulfate (Amresco, Solon, Ohio, USA), 1 mM EDTA and protease inhibitor were added. After the samples were placed on ice for 30 min, they were centrifuged in a microcentrifuge (Heraeus Fresco 17 Centrifuge, Thermo Fisher, Germany) for 20 min at 4 °C and 12,000 rpm. The tubes were gently removed from the centrifuge and placed on ice. The supernatant was aspirated and placed in a fresh tube kept on ice, and the pellet was discarded. Under reducing conditions, proteins (30 μg) were resolved through 12% sodium dodecyl sulfate–polyacrylamide gel electrophoresis. The gel was first equilibrated in transfer buffer (Tris 250 mM; Glycine 1.92 M) (Omics Bio, Taipei, Taiwan) and then placed in the “transfer sandwich” (filter paper-gel-membrane-filter paper), cushioned by pads and pressed together by a support grid. The supported gel sandwich was placed vertically in a tank between stainless steel/platinum wire electrodes and the tank was filled with transfer buffer. Multiple gels electrotransferred were performed used high voltage (200 V) for an hour in the room temperature and tank transferred onto a polyvinylidene difluoride membrane (ImmobilonP, Millipore, Bedford, MA, USA). The membranes were blocked with 5% non-fat dried milk (ImmobilonP, Millipore) and subsequently incubated with platelet-derived growth factor-A (PDGF-A, 1:750, E10 Sc-9974, Santa Cruz Biotechnology, CA, USA), PDGF-B (1:750, F3 Sc-365805, Santa Cruz Biotechnology), and anti-β-actin (1:1,000, C4 sc-47778, Santa Cruz Biotechnology). They were then incubated with horseradish peroxidase–conjugated goat antimouse (Pierce Biotechnology, Rockford, IL, USA). The protein bands were detected using the BioSpectrum AC System (UVP, Upland, CA, USA) and VisionWorks LS Software version 8.6 (UVP, Upland, CA, USA) with acquisition mode WB HS Top (Image Integration – Total Time, 2s exposure time, 50 number of frames).

### Metabolomics

#### Sample preparation

The lung homogenate samples were extracted using 100 μl of methanol solution (Macron Chemicals, Center Valley, PA, USA) and H_2_O (Cat # W4502, Sigma-Aldrich, St. Louis, MO, USA; 7:3, v:v). After two freeze–thaw cycles, the samples were vortexed. After each sample was centrifuged (15 min at 4 °C and 12,000 × *g*), dried in a speed vacuum, and resuspended in 0.3 ml of 50:50 H_2_O/CH_3_CN, the supernatant was recovered.

#### Liquid chromatography

A chromatographic separation was performed using a Waters Acquity ultra performance liquid chromatography (UPLC) system (Waters, Milford, MA, USA). A UPLC BEH C18 guard column (1.7 μm, 5 mm) was used as the reverse-phase column, and the analytical column (1.7 μm, 2.1 × 100 mm) was maintained at 45 °C. The linear gradient separations used mobile phases composed of (A) water containing 0.1% formic acid and (B) acetonitrile containing 0.1% formic acid. The gradient profile was as follows: 0–1 min, 1% B; 1–15 min, 1–100% B;15–17 min, 100% B; 17–17.1 min, 100–1% B; 17.1–20 min, 1% B. The eluent was then injected directly into the mass spectrometer, without being divided first.

#### Mass spectrometry

Mass spectrometry (MS) was performed using a SYNAPT G2 quadrupole time-of-flight mass spectrometer (Waters MS Technologies, Manchester, UK). The machine was running in positive mode. For the detection of positive ionization mode, the parameters of the mass spectrometer were set as follows: desolvation gas flow rate: 900 L/h; desolvation temperature: 550 °C; cone gas flow rate:15 L/h; source temperature: 120 °C; capillary voltage: 2.8 kV; cone voltage: 40 V; and TOF MS scan range: 50 to 1,000 *m*/*z*. The Waters MSE acquisition mode was used with a data acquisition rate and an interscan latency of 1.2 and 0.02 s, respectively, and the full exact masses were recorded simultaneously through rapid cycling between two functions. The first function collected data with low-impact energies of 4 eV for the collision cell trap and 2 eV for the collision cell transfer, whereas the second collected data with a transfer collision energy ramp from 15 to 25 eV. All the analyses were performed using a lockspray to assure accuracy and reproducibility. Leucine-enkephalin (*m*/*z* = 556.2771) at a concentration of 1 ng/μL and a flow rate of 5 μL/min was used as the lockmass. The lockspray frequency was set to 20 s, and the data were collected in continuous mode. Waters MassLynx mass spectrometry software (version 4.1) was used for all data collection.

#### Metabolomic data analysis

**Figure 1:**
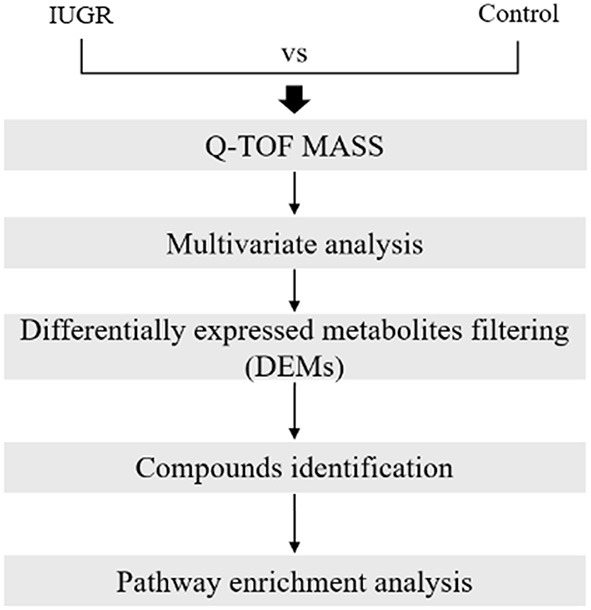
Metabolomic data analysis workflow. The workflow included a discrete combination of quadrupole time-of-flight mass spectrometry, multivariate statistics, multivariate machine learning, filtering of differentially expressed metabolites, compound identification, and pathway enrichment analysis.

The metabolomic data analysis involved mass peak detection, multivariate analysis, differentially expressed metabolite (DEM) filtering, compound identification, pathway-related compound discovery, and pathway enrichment analysis (Figure 1). Progenesis QI v.2.1 was used to import, process, normalize, and review raw UPLC-MS/MS data (Non-linear Dynamics, Newcastle, UK). The DEMs were identified using a cutoff value equating to a ≥1.2-foldchange in median intensity between the two groups of samples. Progenesis QI was used for compound identification by comparing the data against the human metabolome database, and the compound prediction had an overall score of 40 according to mass accuracy and isotope patterns. A cut off value that was ≥ 36 of the identified score was applied. The pathway-related compounds were compared against the Kyoto Encyclopedia of Genes and the Human Metabolome Database, or HMDB (https://hmdb.ca), and underwent pathway enrichment analysis performed using MetaboAnalyst. All full MS sample was obtained for quality control pool reference, with ≥ 90% alignment with the reference, indicating the reliability of the ACQUITY™ Premier CSH Phenyl-Hexyl 1.7 μm column separation approach. Unique ions (retention time and *m*/*z* pairs) were pooled (obtaining the sum of the abundancies of the unique ions) to generate unique “features” (retention time and *m*/*z* pairs) typical of unannotated metabolites using both adduct and isotope deconvolutions. The data were normalized to all the features with Progenesis QI. Each feature ion was measured in each run, allowing for the calculation of the ratio of the abundance of feature ions in a given run to the corresponding value in the normalized reference. Log10 transformation was performed using Progenesis QI to produce a normal distribution of all the ratio data in each process for all the samples, and the subsequent scalar estimation shifted the log10 distribution to a normalized reference. The FMS data were then used for relative quantification. For each feature across both sample groups, the minimum percent coefficient of variance was calculated. Prior to statistical tests of significance, the data was exported to EZ Info (Umetrics Software), and unsupervised (percent of mean). PCA was conducted to visualize the clustering of the data groups (with all features included). Furthermore, within Progenesis QI, a one-way ANOVA test was used to assess significance between IUGR and control groups, returning a *p*-value for each feature (retention time m/z descriptor), with a nominal *P*-value of 0.05 considered significant.

### Statistical analysis

The data are presented as means ± standard deviations (SDs). Differences were considered statistically significant when the *p* value was < 0.05. A one-way ANOVA was conducted using Progenesis QI to identify significant differences between the IUGR and control groups. The fold change (FC) threshold calculated by Progenesis QI from the combined abundance data was used to further filter significant features, with a FC of ≥ 1.2 considered significant (13–15). Volcano plots were used to represent dysregulated metabolites [log2 [FC] vs. –log10 [*p* value]]. Within Progenesis QI, tentative and putative annotations were determined using accurate mass measurements (<5 ppm error), isotope distribution similarity, and manual assessment of fragmentation spectrum matching (when applicable) conducted by searching the Human Metabolome Database (16), Metlin (17), MassBank (18), and the National Institute of Standards and Technology database (19). MetaboAnalyst 5.0 was used to perform multivariate analysis. Pathway enrichment and metabolite pathway analysis were performed using annotated feature lists that were statistically significant in the discovery data set (20, 21).

## Results

### UPI rats had lower body weight on postnatal day 7

In total, 39 control pups were delivered by four sham-operated dams, and 26 IUGR rats were delivered by four dams in which UPI was induced. The mean birth weight of the 26 IUGR rats (5.89 ± 0.74 g) was significantly lower than that of the 39 control rats (6.36 ± 0.55 g; *p* < 0.01). On postnatal days 0 and 7, we retrieved 12 and 8 pups from the sham-operated and UPI-induced dams, respectively. Compared with the control rats (*n* = 12), the IUGR rats (*n* = 8) had a significantly lower average body weight on postnatal day 7 (14.50 ± 0.55 g vs. 16.90 ± 1.39 g; *p* < 0.001). The body weight growth velocity was 1.5 and 1.3 g/day in the control and IUGR rats, respectively.

### UPI altered lung development in growth-restricted newborn rats

**Figure 2:**
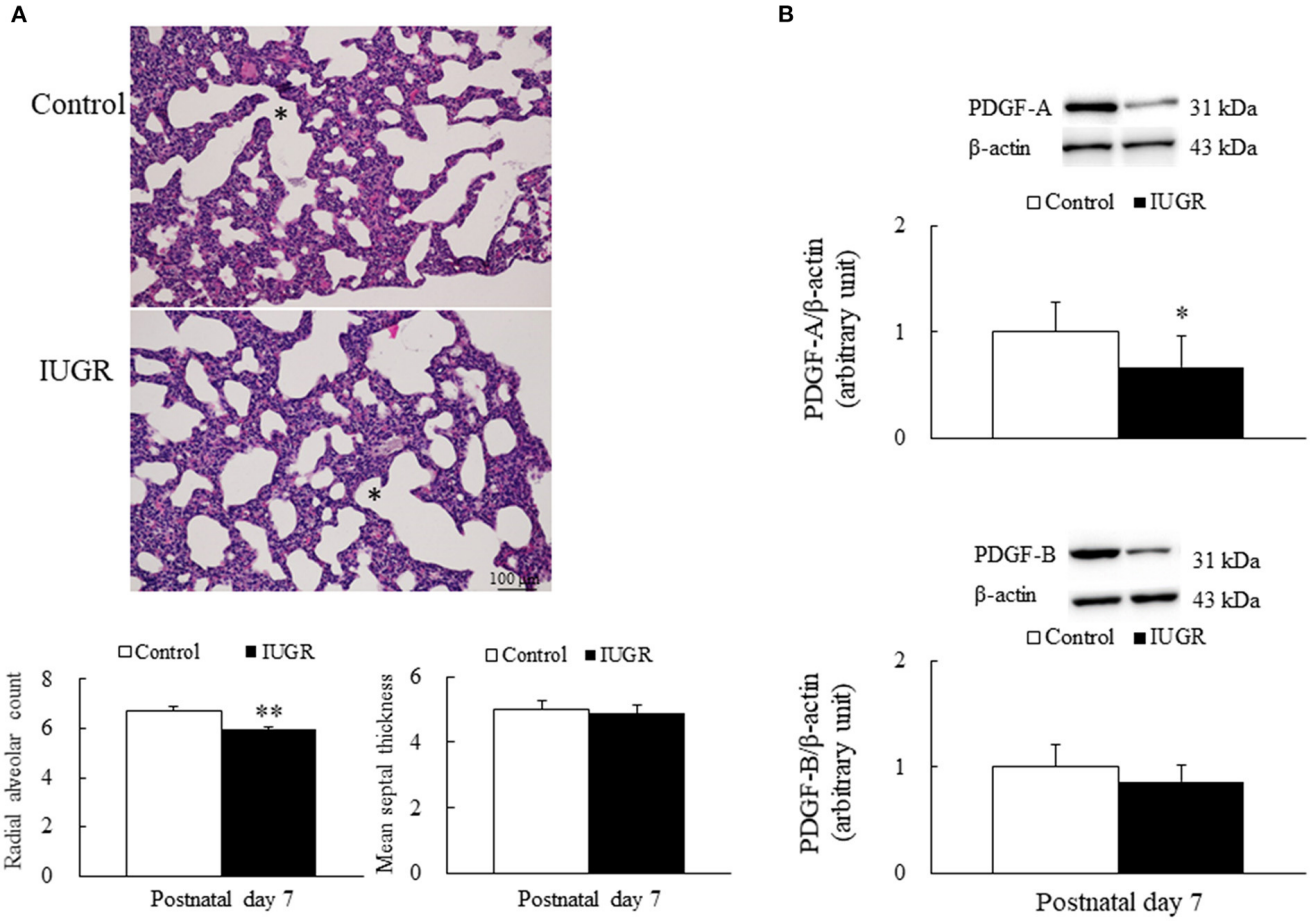
**(A)** Representative lung sections stained with hematoxylin and eosin and radial alveolar count and mean septal thickness **(B)** representative Western blots of PDGF-A and PDGF-B in the lung tissue samples of the control and intrauterine growth restriction (IUGR) groups on postnatal day 7. ^*^Denotes a respiratory bronchiole. Compared with the control rats, the IUGR rats had a significantly higher radial alveolar count, significantly lower average PDGF-A levels, and comparable mean septal thickness and PDGF-B levels. Data are presented as means ± SDs. ^*^p < 0.05, ^**^p < 0.01.

Representative lung sections stained with hematoxylin and eosin are presented in Figure 2A. We measured RAC as an index of alveolar development. The control rats exhibited normal lung morphology, and the IUGR rats had a significantly lower average RAC than the control rats (*p* < 0.01). The MST was comparable between the control and IUGR rats.

### UPI resulted in lower PDGF-A and PDGF-B levels in growth-restricted newborn rats

Representative Western blots and quantitative data determined using densitometry for PDGF-A and PDGF-B are presented in Figure 2B. The IUGR rats had significantly lower PDGF-A levels (*p* < 0.05) and lower PDGF-B levels than the control rats.

### UPI induced a different metabolic profile in growth-restricted newborn rats

**Figure 3:**
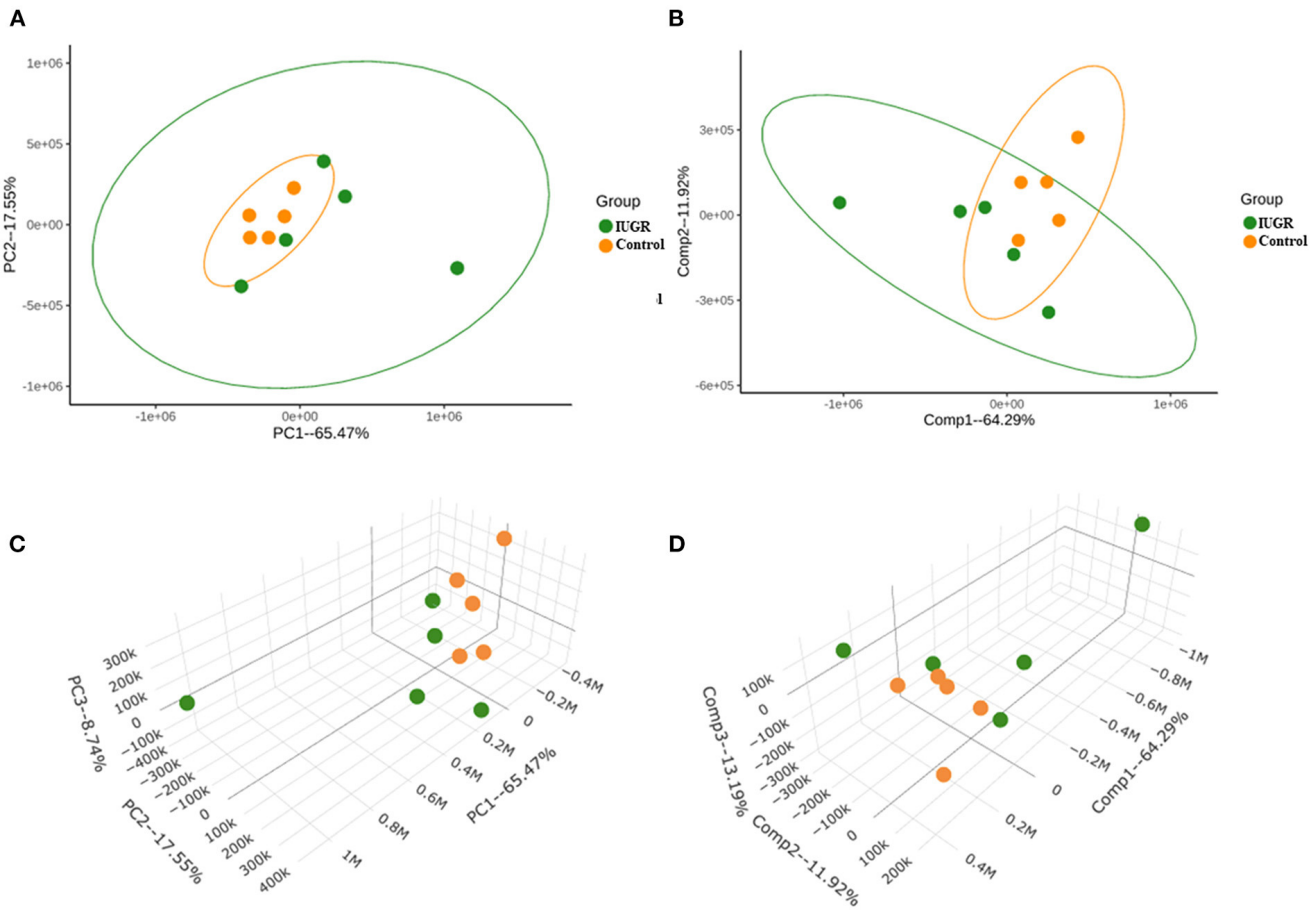
**(A)** Principal component analysis (PCA) score plot of IUGR rat lung tissue samples harvested on postnatal day 7 (in green) relative to the control samples (in orange). **(B)** Partial least squares discriminant analysis (PLS-DA) score plot of the IUGR rat lung tissue samples relative to the control samples. **(C)** Three-dimensional PCA score chart of IUGR rat lung tissue samples harvested on postnatal day 7 (in green) relative to the control samples (in orange). **(D)** Three-dimensional PLS-DA score chart of the IUGR rat lung tissue samples relative to the control samples (*n* = 5).

The PCA method employed in this study, which was based on the ion intensity of 10 significant metabolites in lung tissue samples of the control and IUGR rat pups, was used to further investigate the statistically significant metabolic changes induced by IUGR in the lungs of the IUGR rats. Three-dimensional score charts of the lung tissue samples are presented in Figures 3C,D. The metabolite pattern changed significantly, and the patterns of the IUGR group differed significantly from those of the control group. PCA was performed to give an overview of metabolites data from all samples. In the score plot, each point on the scores plot represents an individual sample. The PCA score plots (Figure 3A) demonstrated some separation between control and IUGR groups, and the separation between controls and IUGR group was especially obvious. The PCA scores plot showed clear separations between the three groups onto the first two principal components (PCs), accounting for 65.47% of the total variance (47.92 and 17.55%, respectively) of the PCA scores plot. Furthermore, the close proximity PCA score of the IUGR and control groups indicated that IUGR began to affect lung tissue metabolism of the IUGR rat pups by postnatal day 7. Partial least squares discriminant analysis (PLS-DA) was performed on the experimental data (Figures 3B,D) since it is an efficient and optimal method used in metabolomics when the number of metabolites detected is high and likely correlated. PLS-DA showed a well-defined separation between groups, being component 1 (64.29%) and component 2 (11.92%) (Figure 3B) which also indicated that the metabolic profile of the IUGR rat pups had changed significantly by postnatal day 7.

**Figure 4:**
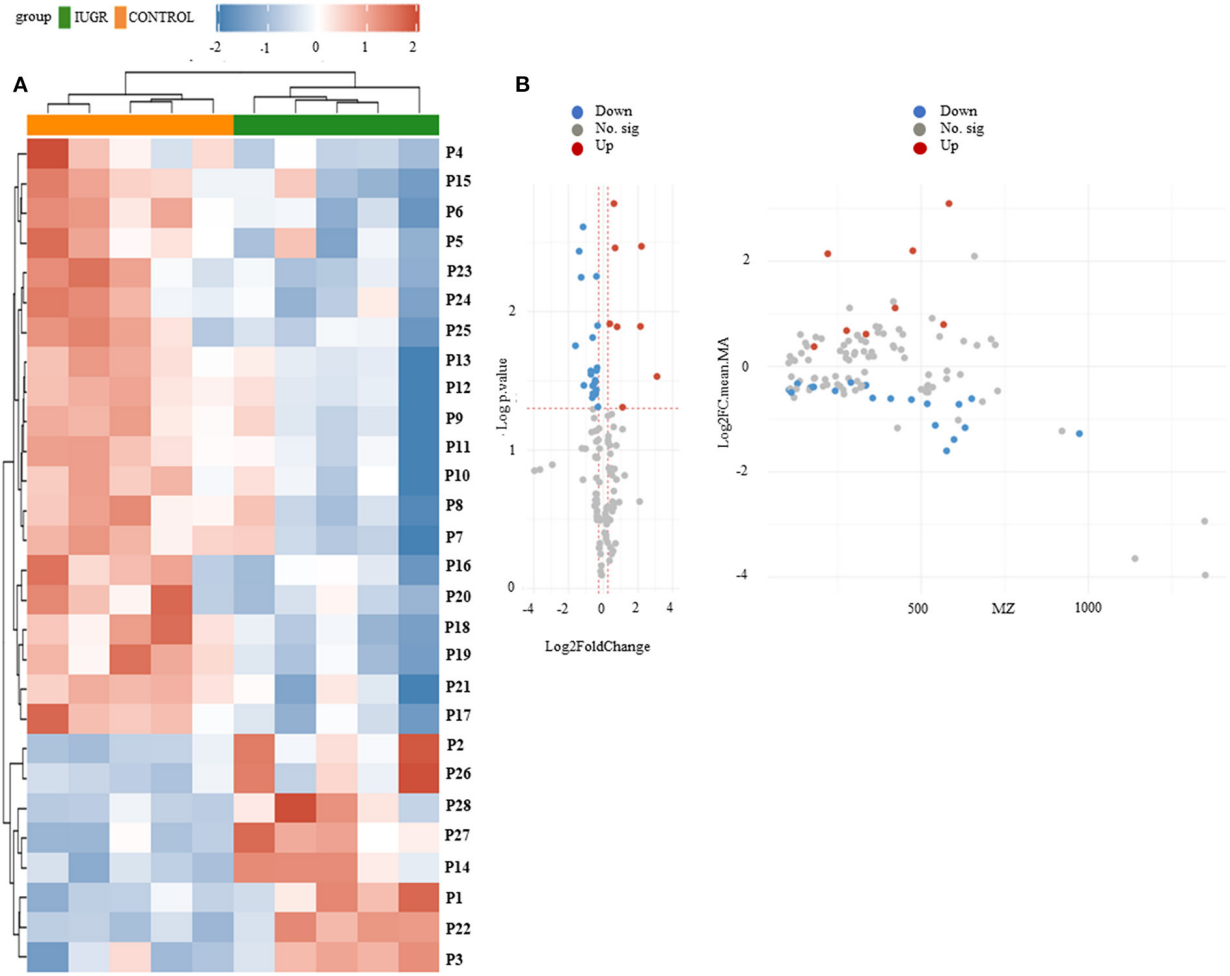
**(A)** Hierarchical clustering analysis and heat map of control and IUGR rat lung tissue samples harvested on postnatal day 7. Color scale represents the scaled abundance of each variable, with red indicating high abundance and blue indicating low abundance. Compounds represented in the heat map are numbered according to their peak numbers (*n* = 5). **(B)** Volcano plots of ultraperformance liquid chromatography–mass spectrometry (MS)/MS datasets. The y-axis represents *p* value converted to –log [*p* value] and the x-axis represents log_2_ [fold change]. Significant metabolites (fold change <1.2, *p* value < 0.05) were highlighted in blue and red. Gray points represent non-significant metabolites.

The heat map of the hierarchical cluster analysis revealed two main clusters that separated the control and IUGR rat lung tissue samples (Figure 4A). The volcano plot incorporated the FC and *p* values to identify significant metabolites. Of the 65 metabolites examined, 10 differed significantly between the groups (*p* < 0.05). The *p* values and descriptions of the significant differential metabolites are presented in Table 1. These findings indicate that IUGR induced metabolic changes such as glutathione; arginine and proline; thiamine; taurine and hypotaurine; alanine, aspartate, and glutamate; cysteine and methionine; glycine, serine, and threonine; glycerophospholipids; and purine and the biosynthesis of aminoacyl-tRNA and of pantothenate and CoA in lung development. Overall, the PCA and hierarchical cluster analysis revealed that the lung tissues of healthy and IUGR newborn rats could be distinguished according to their comprehensive metabolic profiles.

**Table 1:** Major differential metabolites and identified pathways in the rat lungs on postnatal day 7.

	**Initial untargeted UPLC-MS/MS**					
**Pathway**	**Name**	**Formula**	**KEGG ID**	**Mol. Wt**	**Retention Time (mins)**	***p* value**
**Glutathione metabolism**	Pyroglutamicacid	C5H7NO3	C01879	130.049472	2.0872	0.00008
	Cysteinylglycine	C5H10N2O3S	C01419	179.047733	2.0872	0.00031
	L-Cysteine	C3H7NO2S	C00097	243.047239	2.0872	0.00044
**Arginine and proline metabolism**	Pyrrolinehydroxycarboxylicacid	C5H7NO3	C04281	130.049472	2.0872	0.00008
	1-Pyrroline-4-hydroxy-2-carboxylate	C5H7NO3	C04282	130.049472	2.0872	0.00008
	(3R,5S)-1-pyrroline-3-hydroxy-5-carboxylicAcid	C5H7NO3	C04281	130.049472	2.0872	0.00008
**Thiamine metabolism,** **Taurine and hypotaurine metabolism,** **Pantothenate and CoA biosynthesis,** **Cysteine and methionine metabolism,** **Glycine, serine and threonine metabolism,** **Aminoacyl-tRNA biosynthesis**	L-Cysteine	C3H7NO2S	C00097	243.047239	2.0872	0.00044
**Alanine, aspartate and glutamate metabolism**	N-Acetyl-L-asparticacid	C6H9NO5	C01042	176.056002	2.951017	0.00001
**Glycerophospholipid metabolism**	LysoPC[20:5(5Z,8Z,11Z,14Z,17Z)/0:0]	C28H48NO7P	C04230	542.32244	6.4016	0.03953
**Purine metabolism**	Guanosine	C10H13N5O5	C00387	567.177136	1.27715	0.03102

We identified the 65 metabolites by comparing their *m*/*z* values and extracted ion chromatograms against the metabolite database and manually checking for matching *m*/*z* values and peak shapes. We conducted a one-way ANOVA to identify features of which the genotype-dependent abundance differed between the IUGR and control group. The ANOVA revealed 345 features in the pathway, among which 14 were significant. In Figure 4B, volcano plot analysis identified differential metabolites and showed significance and magnitude of change are plotted on the x-axis and y-axis, respectively.

### Metabolic pathway analysis of control and IUGR rat lung tissue samples

**Figure 5:**
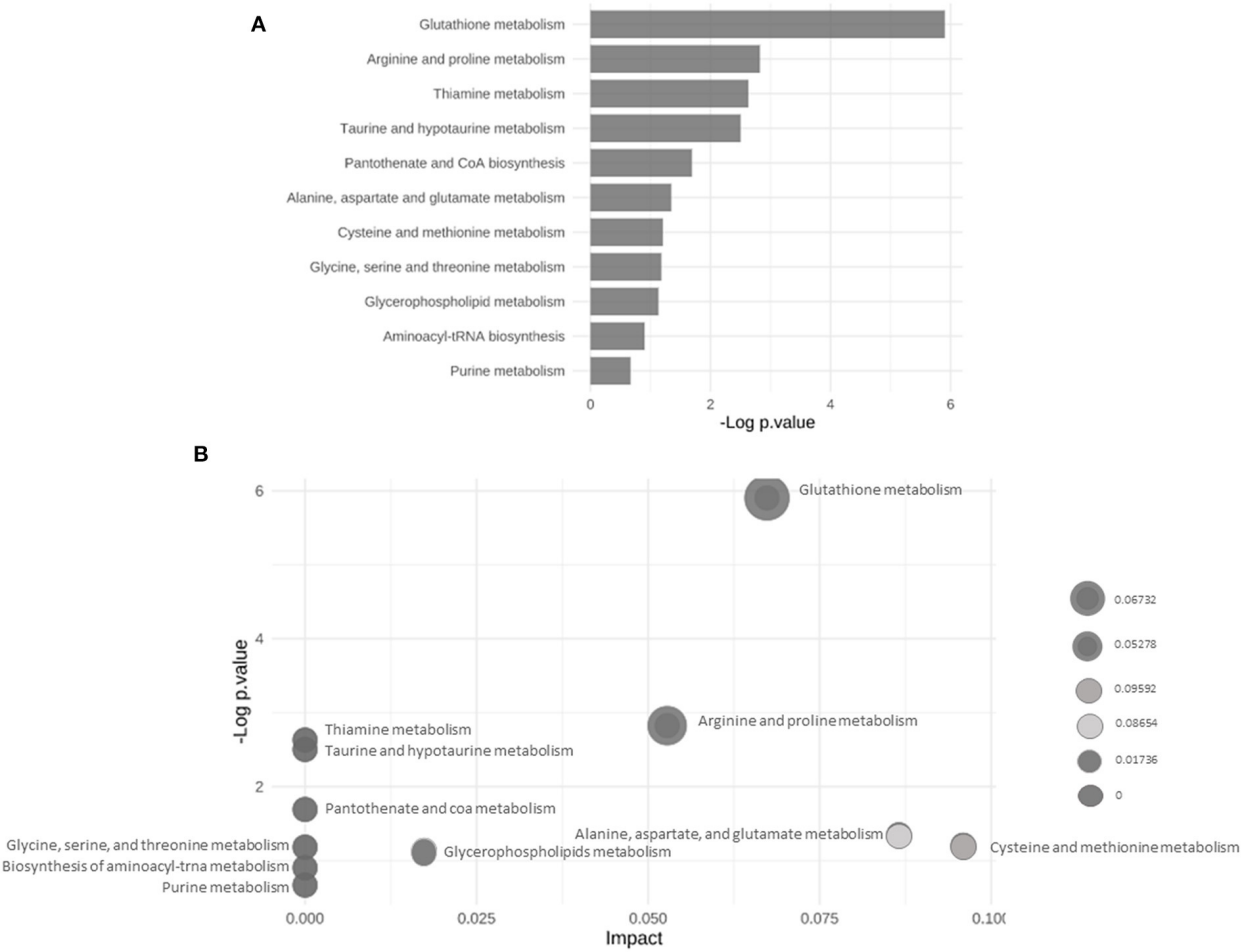
**(A)** Bar chart of the results of the enrichment analyses highlighting the altered metabolic pathways in the control and IUGR rat lung tissue samples harvested on postnatal day 7. **(B)** Bubble plot of the results of pathway enrichment of the control and IUGR rat lung tissue samples harvested on postnatal day 7 (*n* = 5).

**Figure 6:**
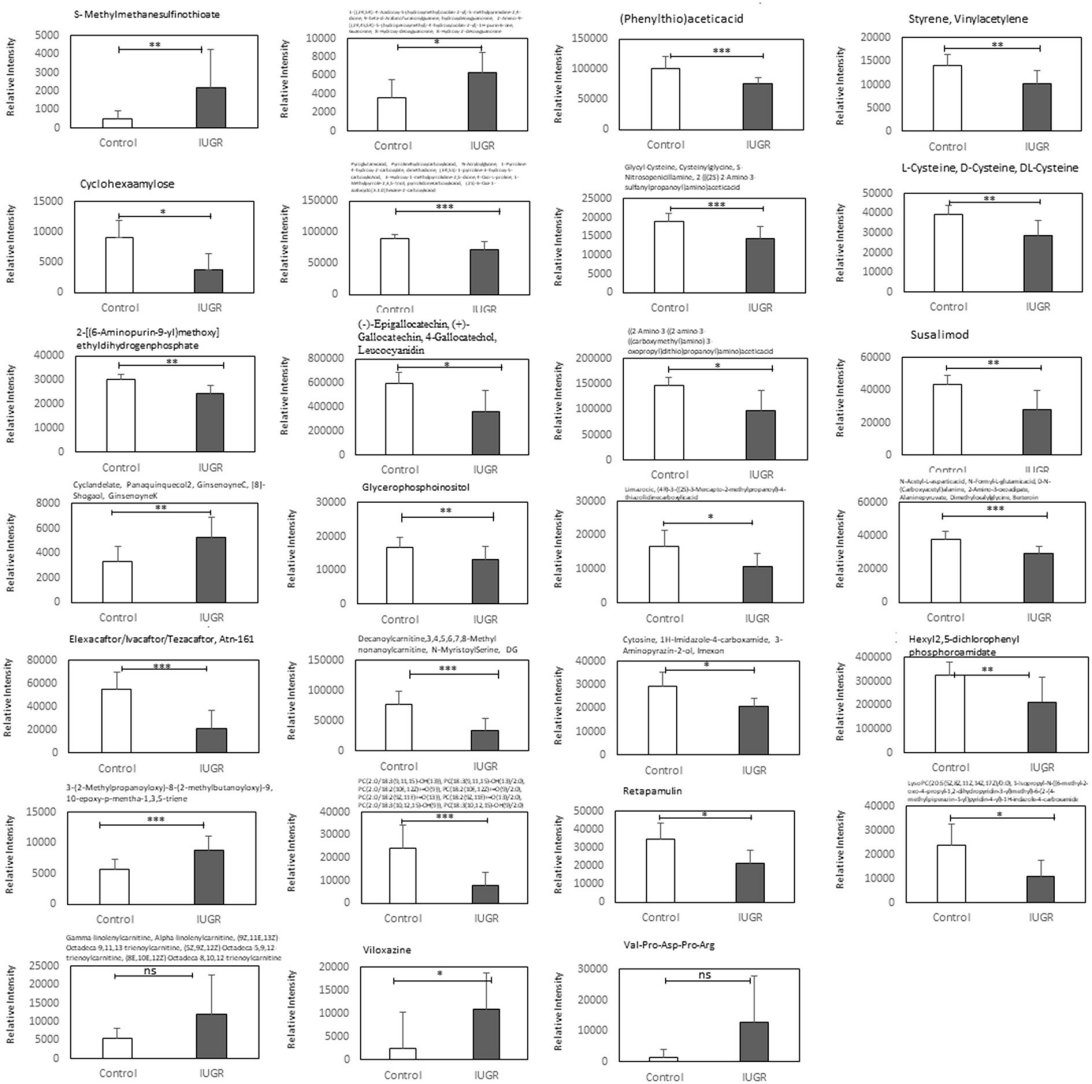
Relative ion intensities of significantly differential metabolites in the control and IUGR rat lung tissue samples harvested on postnatal day 7 (*n* = 5). Data are presented as means ± SDs. ns, not significant. ^*^p < 0.05; ^**^p < 0.01; ^***^p < 0.001.

The main altered metabolic pathways included those related to the metabolism of glutathione; arginine and proline; thiamine; taurine and hypotaurine; alanine, aspartate, and glutamate; cysteine and methionine; glycine, serine, and threonine; glycerophospholipids; and purine and the biosynthesis of aminoacyl-tRNA and of pantothenate and CoA. A total of 11 pathways were identified, among which two differed significantly between the study groups (*p* < 0.05; Figure 5 and Table 2). The signaling compounds identified in the rat lung tissue samples harvested on postnatal day 7 represented their peak numbers (Table 3). Among the main differential metabolites in the IUGR group, the mean levels of P2, P3, P14, P22, and P27were significantly higher than those in the control group. However, the levels of P26 and P28 did not differ significantly between the IUGR group and the control group (Table 3 and Figure 6).

**Table 2:** Network activity prediction analysis of the metabolic pathways.

**Network activity prediction analysis**			
**Pathway name**	**# of significant features within pathway**	**# features within pathway**	***p*** **value**
Glutathione metabolism	3	28	0.0027283
Arginine and proline metabolism	2	38	0.059357
Thiamine metabolism	1	7	0.072041
Taurine and hypotaurine metabolism	1	8	0.081926
Pantothenate and CoA biosynthesis	1	19	0.18434
Alanine, aspartate and glutamate metabolism	1	28	0.26006
Cysteine and methionine metabolism	1	33	0.29923
Glycine, serine and threonine metabolism	1	34	0.30683
Glycerophospholipid metabolism	1	36	0.32179
Aminoacyl-tRNA biosynthesis	1	48	0.40539
Purine metabolism	1	66	0.51287

**Table 3:** Identification of mass spectrometry signals in the rat lungs on postnatal day 7.

**Peak number/ chemical class**	**Identification**
P1	7-Aminomethyl-7-carbaguanine, 9-Ethylguanine
P2	S- Methylmethanesulfinothioate
P3	1-[(2R,5R)-4-Azidooxy-5-(hydroxymethyl) oxolan-2-yl]-5-methylpyrimidine-2,4-dione, 9-beta-d-Arabinofuranosylguanine, hydroxydeoxyguanosine, 2-Amino-9-[(2R,4S,5R)-5-(hydroperoxymethyl)-4-hydroxyoxolan-2-yl]-1H-purin-6-one, Guanosine, 8-Hydroxy-deoxyguanosine, 8-Hydroxy-2-desoxyguanosine
P4	(Phenylthio)aceticacid
P5	Styrene, Vinylacetylene
P6	Cyclohexaamylose
P7	Pyroglutamicacid, Pyrrolinehydroxycarboxylicacid, N-Acryloylglycine, 1-Pyrroline-4-hydroxy-2-carboxylate, dimethadione, (3R,5S)-1-pyrroline-3-hydroxy-5-carboxylicAcid, 3-Hydroxy-1-methylpyrrolidine-2,5-dione, 4-Oxo-L-proline, 1-Methylpyrrole-2,3,5-triol, pyrrolidonecarboxylicacid, (2S)-6-Oxa-1-azabicyclo[3.1.0]hexane-2-carboxylicacid
P8	Glycyl-Cysteine, Cysteinylglycine, S-Nitrosopenicillamine, 2-[[(2S)-2-Amino-3-sulfanylpropanoyl]amino]aceticacid
P9	L-Cysteine, D-Cysteine, DL-Cysteine
P10	2-[(6-Aminopurin-9-yl)methoxy]ethyldihydrogenphosphate
P11	(-)-Epigallocatechin, (+)-Gallocatechin, 4-Gallocatechol, Leucocyanidin
P12	[(2-Amino-3-((2-amino-3-((carboxymethyl)amino)-3-oxopropyl)dithio)propanoyl)amino]aceticacid
P13	Susalimod
P14	Cyclandelate, Panaquinquecol2, GinsenoyneC, (8)-Shogaol, GinsenoyneK
P15	Glycerophosphoinositol
P16	Limazocic, (4R)-3-((2S)-3-Mercapto-2-methylpropanoyl)-4-thiazolidinecarboxylicacid
P17	N-Acetyl-L-asparticacid, N-Formyl-L-glutamicacid, D-N-(Carboxyacetyl)alanine, 2-Amino-3-oxoadipate, Alaninepyruvate, Dimethyloxalylglycine, Berteroin
P18	Elexacaftor/Ivacaftor/Tezacaftor, Atn-161
P19	Decanoylcarnitine,3,4,5,6,7,8-Methylnonanoylcarnitine, N-MyristoylSerine, DG
P20	Cytosine, 1H-Imidazole-4-carboxamide, 3-Aminopyrazin-2-ol, Imexon
P21	Hexyl2,5-dichlorophenylphosphoroamidate
P22	3-(2-Methylpropanoyloxy)-8-(2-methylbutanoyloxy)-9,10-epoxy-p-mentha-1,3,5-triene
P23	PC(2:0/18:3(9,11,15)-OH(13)), PC(18:3(9,11,15)-OH(13)/2:0), PC(2:0/18:2(10E,12Z)+=O(9)), PC(18:2(10E,12Z)+=O(9)/2:0), PC(2:0/18:2(9Z,11E)+=O(13)), PC(18:2(9Z,11E)+=O(13)/2:0), PC(2:0/18:3(10,12,15)-OH(9)), PC(18:3(10,12,15)-OH(9)/2:0)
P24	Retapamulin
P25	LysoPC(20:5(5Z,8Z,11Z,14Z,17Z)/0:0), 1-Isopropyl-N-((6-methyl-2-oxo-4-propyl-1,2-dihydropyridin-3-yl)methyl)-6-(2-(4-methylpiperazin-1-yl)pyridin-4-yl)-1H-indazole-4-carboxamide
P26	Gamma-linolenylcarnitine, Alpha-linolenylcarnitine, (9Z,11E,13Z)-Octadeca-9,11,13-trienoylcarnitine, (5Z,9Z,12Z)-Octadeca-5,9,12-trienoylcarnitine, (8E,10E,12Z)-Octadeca-8,10,12-trienoylcarnitine
P27	Viloxazine
P28	Val-Pro-Asp-Pro-Arg

## Discussion

In this study, we examined IUGR rats on postnatal day 7 because we found that UPI-induced IUGR rats exhibited a significantly higher volume fraction of alveolar airspace on postnatal day 7 compared with the control rats (10). We used a non-targeted metabolomic approach to elucidate the effects of IUGR on fetal lung development and to identify metabolites that may serveas perinatal biomarkers of IUGR-associated abnormal lung development. Our *in vivo* model demonstrated that the induction of IUGR through bilateral uterine artery ligation in the pregnant rats altered the lung development of the rat pups, as evidenced by the average RAC of the IUGR group being significantly lower than that of the control group. These findings are consistent with clinical observations of neonates with IUGR, in which BPD has been linked to long-term functional impairment due to abnormal lung growth (22). IUGR pups had significantly lower levels of PDGF-A and PDGF-B than control pups, according to western blot. This finding is related to the mechanism of endothelial cell proliferation inhibition leading to decreased alveolar septation; this effect may be due to trophic effects on the vascular endothelium, altered production of endothelial-derived products such as nitric oxide or PDGF (23).

This study provides supporting evidence and clinically relevant information regarding the effects of IUGR on rat fetuses, are expected to reproduce the physiological features of IUGR in humans. Targeted metabolic profiling in the IUGR rat model revealed that IUGR causes statistically significant metabolic changes in the fetal rat lung. These metabolic changes included changes in the ion intensity of amino acids, fatty acids, and energy metabolism intermediates. Our findings regarding altered pulmonary metabolites may facilitate the identification of clinical biomarkers for the early prenatal diagnosis of IUGR and impaired lung development as well as the identification of several possible treatment targets. IUGR has been determined to affect alveolarization in the lungs of animals in previous studies; therefore, models of IUGR are occasionally utilized as a BPD model as well (24, 25). In this study, we evaluated metabolic changes in the lungs of the IUGR rats by postnatal day 7 through a metabolic analysis (*n* = 5). The metabolites that differed significantly between the groups (*p* < 0.05 and FC ≥ 1.2) were selected and subjected to pathway analysis. A PLS-DA score plot revealed that the clustering differed significantly between the control and IUGR groups, suggesting that metabolic changes occurred in the lungs of the IUGR rats (Figure 4 and Table 3).

The 10 metabolites that differed significantly between the groups are summarized in Table 1. Glutathione is a key antioxidant that protects the body from oxidative stress, and glutathione levels are lower in patients with IUGR than in healthy individuals (26). Higher glutathione consumption and disrupted glutathione synthesis have both been linked to IUGR. In one study, the total (reduced, oxidized, and protein-bound) levels of glutathione in preeclamptic pregnancies were lower than that in normotensive pregnancies (27). This indicates that extrauterine and intrauterine factors, rather than genetics, are responsible for IUGR. Cysteine availability affects the rate of glutathione production. Glutathione is required for cell development regulation, and a lack of glutathione may result in severe mucosal damage, including epithelial cell destruction and mitochondrial degeneration (28). Another study discovered that glutathione is an essential molecule in redox cellular signaling because it regulates the oxidation of cysteines from oxidative stress sensors. However, inactivation of enzymes with cysteine is the most basic redox signaling mechanism. One such example is the stimulation of phosphorylation pathways by oxidative inactivation of phosphatases and it may be linked to IUGR (29, 30). The pathophysiology of illness in newborns is thought to be dominated by oxidative damage caused by oxygen toxicity (31, 32). This problem is aggravated in newborns with IUGR, which causes oxidative damage in the lungs (33). Previous studies have revealed that oxidative stress plays an important role in the development of IUGR (34–36). N-acetylcysteine is a precursor to the amino acid cysteine, which has two key metabolic functions. By contributing to glutathione production, cysteine participates in the general antioxidant activities of the body. By modulating the glutamatergic system, cysteine influences the reward/reinforcement pathway (37). A study involving a rat model of preeclampsia and fetal growth restriction revealed that N-acetylcysteine can alleviate maternal hypertension (38). N-acetylcysteine also effectively protects against chronic intrauterine hypoxia in animals (39). Chronic fetal hypoxia is the leading cause of IUGR as well as fetal and neonatal morbidity and mortality (40–42).

L-arginine, a nutritionally essential amino acid for fetuses (43), is a precursor for nitric oxide synthesis (44). Arginine concentrations increase with age (45). Consequently, L-arginine may play a vital role in fetal nutrition and oxygenation, resulting in the alleviation of the symptoms of IUGR, higher birth weights, and a decreased risk of neonatal morbidity and mortality (43). Furthermore, elevated proline and alanine levels were also observed throughout the first week of life (45).

In another study, elevated phenylalanine levels are associated with lung disease, and significant increases in phenylalanine can be observed in infants with respiratory distress syndrome (46). Urinary concentrations of alanine and phenylalanine are lower in infants with IUGR than in healthy infants, indicating that amino acid metabolism is often disrupted early in the pregnancy. Elevated levels of phenylalanine and alanine during pregnancies have been observed in several studies (47–49). The umbilical cord blood concentrations of phenylalanine and alanine of infants with IUGR are also lower than those of healthy infants, indicating that phenylalanine and alanine can be used to differentiate between fetuses with IUGR and healthy controls during healthy pregnancies. This maybe because of changes in placental tissue due to the hypercatabolic state associated with IUGR (46). Thiamine deficiency induces IUGR in rat fetuses, specifically when induced by a thiamine-deficient diet plus pyrithiamine supplementation (50). Taurine is involved in various biological processes, including apoptosis, cell volume regulation, neuromodulation, antioxidant defense, protein stabilization, and stress responses (51). The levels of placental amino acid transporters, which transport taurine and induce the transport of neutral amino acids like glutamate and glycine, are significantly reduced by IUGR during pregnancy (52).

Another metabolite of interest in the present study was methionine, which was altered in several studies. UPI increases methionine levels in IUGR. Methionine is a sulfur amino acid with biological functions, including functions related to protein metabolism, methylation, cysteine production, glutathione reduction, and antioxidant systems (53–55). Phenylalanine and methionine differ considerably, and a cutoff value for phenylalanine that provided excellent differentiation between IUGR and appropriate-for-gestational-age neonates was identified in a previous study (56). Roecklein et al. (50) proposed that thiamine deficiency could induce IUGR in rats. They discovered that thiamine deficiency causes decreases in body weight, placental weight, and liver weight and that a larger brain-to-liver ratio is a sign of IUGR. The average thiamine levels of infants with severe IUGR are significantly lower than those of infants with normal birthweights. In cases of severe IUGR, a modest reduction in plasma thiamine is often observed toward the end of the pregnancy (>36 weeks). These findings suggest that thiamine deficiency maybe a cause of IUGR (57).

Glycine is the main agonist of glycine receptors, which are chloride channels that hyperpolarize the cell membranes of inflammatory cells such as macrophages and neutrophils, thereby desensitizing them to proinflammatory stimuli. In addition, glycine has cytoprotective properties, improves endothelial function, and reduces platelet aggregation. Glycine plays a crucial role in the development of infants' lungs (47, 49).

Prenatal nicotine exposure caused the metabolomic changes of maternal plasma, fetal plasma, and amniotic fluid in nicotine-induced UGR rat model (58). However, the effects of UPI on offspring lung metabolomics were mostly unknown in IUGR offspring. In this study, UPI alters lung development and metabolomics in growth-restricted newborn rats. These findings may clarify metabolic mechanisms underlying IUGR-induced altered lung development. The limitation of this study is that our study only focused on the lungs of IUGR and the control group which only represents knowledge about itself and does not use other organs that allow to study certain conditions in the lungs.

## Conclusion

UPI alters lung development and metabolomics in growth-restricted newborn rats. Our findings may elucidate the metabolic mechanisms underlying IUGR-induced altered lung development and serve as a reference for the development of strategies to prevent and treat altered lung development induced by IUGR.

## Data availability statement

The original contributions presented in the study are included in the article/supplementary material, further inquiries can be directed to the corresponding author.

## Ethics statement

The animal study was reviewed and approved by the Laboratory Animal Care Committee of Taipei Medical University, and the experimental procedures were performed in accordance with the committee's guidelines (LAC-2022-0120).

## Author contributions

C-MC contributed to the conception and design of this study. MY, Z-HH, H-CC, and C-MC contributed to the performance of the experiments, analysis and interpretation of the data, and approval of the manuscript. All authors agree to assume responsibility for all aspects of the work to ensure proper investigation and resolution of issues related to the accuracy or integrity of any part of the work. All authors contributed to the article and approved the submitted version.

## Funding

This work was supported by a grant from Taipei Medical University Hospital (111TMUH-MOST-15; Taipei, Taiwan).

## Conflict of interest

The authors declare that the research was conducted in the absence of any commercial or financial relationships that could be construed as a potential conflict of interest.

## Publisher's note

All claims expressed in this article are solely those of the authors and do not necessarily represent those of their affiliated organizations, or those of the publisher, the editors and the reviewers. Any product that may be evaluated in this article, or claim that may be made by its manufacturer, is not guaranteed or endorsed by the publisher.
